# Investigating Influences of Medial Olivocochlear Efferent System on Central Auditory Processing and Listening in Noise: A Behavioral and Event-Related Potential Study

**DOI:** 10.3390/brainsci10070428

**Published:** 2020-07-04

**Authors:** Aparna Rao, Tess K. Koerner, Brandon Madsen, Yang Zhang

**Affiliations:** 1 Department of Speech and Hearing Science, Arizona State University, Tempe, AZ 85287, USA; 2 VA RR & D National Center for Rehabilitative Auditory Research, Portland, OR 97239, USA; Tess.koerner@va.gov (T.K.K.); brandon.madsen@va.gov (B.M.); 3 Department of Speech-Language-Hearing Sciences & Center for Neurobehavioral Development, University of Minnesota, Minneapolis, MN 55455, USA

**Keywords:** event-related potential (ERP), medial olivocochlear (MOC) efferents, otoacoustic emissions inhibition, contralateral acoustic stimulation (CAS), MMN, P300

## Abstract

This electrophysiological study investigated the role of the medial olivocochlear (MOC) efferents in listening in noise. Both ears of eleven normal-hearing adult participants were tested. The physiological tests consisted of transient-evoked otoacoustic emission (TEOAE) inhibition and the measurement of cortical event-related potentials (ERPs). The mismatch negativity (MMN) and P300 responses were obtained in passive and active listening tasks, respectively. Behavioral responses for the word recognition in noise test were also analyzed. Consistent with previous findings, the TEOAE data showed significant inhibition in the presence of contralateral acoustic stimulation. However, performance in the word recognition in noise test was comparable for the two conditions (i.e., without contralateral stimulation and with contralateral stimulation). Peak latencies and peak amplitudes of MMN and P300 did not show changes with contralateral stimulation. Behavioral performance was also maintained in the P300 task. Together, the results show that the peripheral auditory efferent effects captured via otoacoustic emission (OAE) inhibition might not necessarily be reflected in measures of central cortical processing and behavioral performance. As the MOC effects may not play a role in all listening situations in adults, the functional significance of the cochlear effects of the medial olivocochlear efferents and the optimal conditions conducive to corresponding effects in behavioral and cortical responses remain to be elucidated.

## 1. Introduction

Human auditory perception depends on elaborate neural coding of acoustic properties of the target sounds and involves bidirectional interactions of the afferent and efferent systems along the auditory pathway. The neural coding of auditory stimulation begins in the afferent system of the cochlear inner hair cells on the basilar membrane, which is regulated by the active mechanical behavior of outer hair cells under the control of a complex efferent innervation system originating from the medial olivary complex in the brainstem. There has been considerable discussion regarding the role of the medial olivocochlear (MOC) efferents in auditory perception [1,2]. A major function of the MOC efferents is thought to be the enhancement of transient signals in noise. However, it remains unclear how the activation of the MOC system by ipsilateral and/or contralateral stimulation contributes to the enhanced central processing of the auditory target either autonomously or via interactions with attentional control. The present electrophysiological study was designed to address this question by selecting listening conditions known to be conducive to MOC activation and investigating how it affects cortical-level responses in passive and attentive listening tasks.

Descending efferent neurons from the MOC system synapse on cochlear outer hair cells (OHCs), the source of the cochlear amplifier, and influence their function. The MOC efferents may be activated by the corticofugal system, which extends from the cortex toward subcortical nuclei [3], or by acoustic stimulation of the ear [4]. The MOC efferents exert control in the cochlea by inhibiting OHC activity, thereby reducing basilar membrane vibration. This inhibition occurs in quiet [5,6,7] and in the presence of background noise, leading to reductions in neural fiber responses to noise and enhancing neural responses to transient signals [8,9,10]. This is referred to as the “antimasking effect” or “unmasking effect” [6,11]. The efferent “antimasking” effect is thought to have important implications for the detection of tones and tonal complexes in noise [12,13,14]. Other important impacts of the MOC system arise from its role in the process of learning [15,16,17,18] and its interactions with higher cognitive functions such as attention [17,19]. MOC inhibition may also have a protective effect in the cochlea by reducing the impact of acoustic trauma [20,21].

The beneficial link between MOC inhibition and speech perception remains controversial [15,22,23,24,25,26,27,28]. One complication here is that behavioral results and higher cognitive processing such as speech perception are influenced by several factors [15,22]. For instance, Mishra and Lutman [25] suggest that although the “unmasking” effect may be documented for speech stimuli, it may not be related to MOC effects measured under artificial conditions. In particular, the MOC system may not be used in natural listening conditions. Age may also play a role, with young children being more dependent on the efferent system for listening in noise compared with older children (>9 years) and adults [29]. The MOC-mediated mechanisms in central auditory processing may be recruited only in some specific conditions that remain to be investigated [25].

Auditory event-related potentials (ERPs) provide an avenue for investigating the neurophysiological underpinnings of behavioral effects and attentional processing, which influence the perception of complex signals in noise. ERPs are non-invasive recordings of electrical voltages created due to synchronous post-synaptic cortical activity time-locked to an auditory stimulus. ERPs reflect specific sensory and/or cognitive processes [30]. Specific ERPs that may be used to study the perception of signals in noise are the N1, P2, mismatch negativity (MMN) and P300 responses [31,32,33,34,35,36,37,38,39,40]. Of particular interest to the current study are the MMN and P300 components. MMN occurs as a negative wave in the frontal region of the brain and is elicited when there is a change in a repeated auditory signal. Typically, an oddball paradigm is used with “standard” stimuli occurring repeatedly and “deviant” or target stimuli randomly interspersed. The MMN occurs even when subjects are not attending to stimuli and thus represents an automatic change-detection process. The MMN is sensitive to small stimulus changes and seems to correlate with behavioral discrimination thresholds in normal subjects as well as in clinical populations [41]. By contrast, the P300 is a positive waveform, which peaks around 300 ms and is enhanced when subjects attend to and detect deviants. The P300 is associated with the cognitive processes of stimulus evaluation and categorization. The amplitude of P300 is proportional to the amount of attentional resources required for stimulus processing [42], with latency reflecting stimulus evaluation [43].

In this exploratory study, we used cortical potentials to study the consequences of MOC activation. MOC efferent effects in humans can be measured noninvasively using spontaneous otoacoustic emissions (OAEs) [44] or evoked OAEs [19,25,45,46,47,48], which are by-products of the cochlear amplifier mechanism. The MOC activity measured in one ear may be influenced by incoming sound at either ear, due to ipsilateral, contralateral and bilateral brainstem circuits. The most widely used procedure is to compare the amplitudes of click-evoked OAEs with and without contralateral broadband noise, also called “contralateral acoustic stimulation” (CAS), to document the reduction in the OAE amplitude when CAS is introduced. This contralateral inhibition of OAEs or the “MOC reflex” [49] has been used as a measure of MOC inhibition strength. For the present study, the transient-evoked otoacoustic emissions (TEOAEs) were recorded with and without CAS to document the MOC reflex in participants. This is also the most frequently used protocol for measuring the effects of the MOC reflex [11,12,13,14,15,16,17]. The ERP tasks for our study were designed with and without CAS to parallel the OAE paradigm. ERPs were obtained in an active P300 experimental task and a passive MMN task. Signals were embedded in ipsilateral noise; contralateral noise was used to elicit a greater MOC effect. All the stimulus levels and characteristics were chosen to optimize the recruitment of MOC efferent effects.

Our study was built upon two recent speculations: (1) individuals may not be using the MOC unmasking effect for listening in all speech-in-noise situations, and (2) the cochlear effects of MOC function may not be reflected in tasks that require higher-level processes such as attention. We explored the links between MOC function and listening in noise by obtaining combined electrophysiological and behavioral measurements while stimulating the MOC system. If target recognition is facilitated by the efferent unmasking effect, the modulation of the MMN would be expected in the non-attentive listening condition. In the active listening condition, however, the unmasking effect would not necessarily be reflected in the P300 for attention-related processing. Traditional word recognition in noise testing was also completed for the same participants for comparison.

## 2. Materials and Methods

### 2.1. Participants and Qualification Testing

Data obtained from eleven individuals (10 females, mean age of all participants = 22.3 years, SD = 1.8 years, range = 19–26 years) were analyzed. The data obtained from two participants in addition to the eleven could not be included for analysis due to the noisy measurements obtained on one or more physiological tests (OAEs and ERPs). A negative history of speech, language or cognitive difficulties was established through case history interview. The Human Subjects’ Protection Program at the University of Minnesota approved the study (ethical research approval code: 1308M41281), and all the participants provided informed consent. The participants were scheduled for two 2-hr sessions and were paid for their time. The first session consisted of a comprehensive audiologic examination, including otoscopy, pure-tone air and bone conduction testing, speech reception threshold testing, and word recognition testing using the Central Institute for the Deaf (CID) W-22 word lists. Participants were tested behaviorally on nonsense word recognition. Otoacoustic emissions were also recorded during the first session. The second session consisted of ERP recordings in both passive and active conditions to record the MMN and P300 ERPs, respectively.

All the participants were right-handed as determined by the Edinburgh Handedness Inventory [50]. The participants had hearing within normal limits as revealed by audiometric thresholds at octave frequencies ranging from 250 to 8000 Hz in both ears. The average thresholds and standard deviations at the different octave frequencies in the two ears are presented in Table 1.

The speech reception thresholds (SRTs) measured using spondees and presented live-voice [51] were consistent with pure-tone averages bilaterally. The word recognition scores (WRSs) obtained with the recorded W-22 lists [52] were excellent (average WRS for right ear = 95.27%, SD = 3.44%; average WRS for left ear = 95.27%, SD = 2.93%) at 30 dB Sensation Level (SL) with reference to the SRT (average right ear = 1.82 dB Hearing Level (HL), SD = 2.52 dB; average left ear = 2.72 dB HL, SD = 2.61 dB). The SRT and WRS results were used as qualification criteria to ensure good test reliability and adequate word recognition abilities in quiet, respectively.

The CAS used to elicit the MOC reflex was broadband and was presented from a portable screening audiometer (Beltone 120, Beltone Electronics Company, Glenview, IL, USA) through an Etymotic Research ER-3A (Etymotic Research Inc, Elk Grove Village, IL, USA) insert headphone. The portable audiometer was calibrated to ANSI-ASA standard S3.6-2010 [53]. The spectrum from 250 to 5000 Hz was within 5 dB of the level at 1000 Hz. The thresholds for the broadband noise (BBN) stimulus were found using the modified Hughson-Westlake procedure. The broadband noise was presented at 30 dB SLwith reference to the behavioral threshold for all conditions using CAS. For all participants, the BBN was between 50 and 55 dBA SPL when measured using a sound level meter. Middle-ear acoustic reflexes were not obtained to the different stimuli used in this experiment due to time and equipment constraints. While this level is below the average middle-ear reflex threshold for young adults [54], it has been used in similar studies [29].

### 2.2. OAEs

Click-evoked OAEs were recorded using the Biologic Scout OAE system (2009) (Natus Medical Incorporated, Mundelein, IL, USA). Clicks were presented at 70 dB peSPL at a presentation rate of 11.72 per second. The responses to 2000 clicks were averaged. The clicks were presented in two conditions in each ear: (1) in quiet and (2) in the presence of CAS (broadband noise) presented at 30 dB SL with reference to the BBN behavioral threshold. The recordings for each listening condition were repeated three times per ear, and the results were averaged. The participants were asked to relax in order to obtain good measurements. The filter range was 800 to 6000 Hz. The responses between 3.5 ms and 16.6 ms were analyzed. The total amplitudes of the click-evoked OAEs were calculated by summing the energy in the fast Fourier transform spectrum between 800 and 4500 Hz. The amplitudes of the click-evoked OAEs were compared between ears and conditions.

### 2.3. Listening Conditions for Word Recognition Testing and Event-Related Potentials

Four listening conditions were used during word recognition testing and the ERP experiments: (1) the stimuli were presented to the right ear with ipsilateral noise at a +10 dB signal-to-noise ratio (SNR) without CAS; (2) the same stimuli were presented to the right ear with the addition of CAS; (3) the stimuli were presented to the left ear with ipsilateral noise at a +10 dB SNR, without CAS; and (4) the stimuli were presented to the left ear with CAS. Therefore, Conditions 2 and 4 were similar to Conditions 1 and 3, respectively, but with CAS. Stimuli were presented to the right ear in Conditions 1 and 2 and to the left ear in Conditions 3 and 4. The conditions were randomized throughout the experiment for each participant. The ipsilateral and contralateral noises used in this study were uncorrelated, to prevent the effects of central masking and to reduce masking level difference. A quiet condition was not used, as the premise of the testing was the perception of signals in noise with and without CAS.

### 2.4. Word Recognition in Noise Test

The Nonsense Syllable Test (NST) [55] was used to evaluate monosyllabic nonsense word recognition. This test was chosen to eliminate the effects of semantic content on word recognition. Participants listened to words using TDH 39P headphones and were instructed to repeat back what they heard from the compact-disc recording. Two research assistants knowledgeable in phonetics scored the phoneme responses and averaged the results for a final score. A different list of 25 words was used for each listening condition. Words were presented to the test ear at 30 dB SL with reference to the speech reception threshold (SRT) a +10 dB SNR through an Aurical diagnostic audiometer (GN Otometrics, Schaumburg, IL, USA). Speech-shaped noise was presented continuously through the second channel of the audiometer to the same ear at a +10 dB SNR. As with the other tests in this protocol, CAS was presented continuously at 30 dB SL with reference to the BBN behavioral threshold.

### 2.5. ERPs

Participants were seated in a comfortable chair in an acoustically and electrically treated booth (IAC systems, Bronx, NY, USA). An oddball paradigm included a pure-tone contrast with frequently repeating 2000 Hz standard stimuli and less frequently occurring 2016 Hz deviant stimuli. The signals were mixed with a white-noise masker presented ipsilaterally at a +10 dB SNR. The signals were presented at 50 dB SL relative to the behavioral detection threshold at 2000 Hz. The signal intensity as measured using a sound level meter was 58 to 62 dB SPL. The deviant of 2016 Hz was chosen based on behavioral pilot experiments in which participants scored 80% accuracy. The pure tone stimuli and masker were created using Sony Sound Forge 10 (version 10, Sony Creative Software, Middleton, WI, USA). The pure tone stimuli were 100 ms in duration and included a 10 ms rise time and 10 ms fall time. The presentation probability was 70% for standards and 30% for deviants. Five blocks of 48 trials were presented with 170 standards and 70 deviants per listening condition. The interstimulus interval between two stimuli was randomized within the range of 900~1100 ms. The stimulus order was randomized within the following two constraints: the deviants must have always been separated by at least two standards, and the first stimulus presented in each block must have been a standard. To measure efferent effects, contralateral broadband noise was presented at 30 dB SL with reference to the BBN behavioral threshold (50 to 55 dB SPL A) using the same portable audiometer used for the OAE and word recognition test protocol.

The same oddball paradigm was used in passive MMN and active P300 conditions. The session always began with the passive MMN blocks to avoid drawing participants’ attention to deviants. In this portion of the experiment, participants were asked to watch a movie of their choice that was played silently with subtitles. In the active P300 task, the participants were asked to press a button to identify the deviant stimulus. Participants were given a short practice block in quiet before being presented with the active listening conditions. Behavioral accuracy and response times were recorded from the participant’s button press responses to calculate the sensitivity index d’ and Criterion [56].

EEG data were collected using the Advanced Neuro Technology (ANT) electroencephalography system and a 64-channel Waveguard cap (ANT Neuro, Enschede, Netherlands) in the Zhang Lab at the University of Minnesota. The impedance of the electrodes was below 15 kΩ. The data were band-pass filtered between 0.016 Hz and 200 Hz and digitized using a sampling frequency of 512 Hz. ERP responses were analyzed off-line using ANT’s Advanced Source Analysis software (version 4.6, ANT Neuro, Enschede, Netherlands). The responses were band-pass filtered off-line between 0.2 Hz and 40 Hz. Trials with potentials exceeding ±50 µV were then rejected. The analysis window was from −100 ms to +600 ms relative to the stimulus onset.

Waveforms were referenced to linked mastoids and analyzed using the peak measures from individual electrodes for amplitude and latency values. The latency ranges used for analysis were 120–350 ms and 200–600 ms for MMN and P300 ERPs, respectively. MMN analysis was conducted for data obtained from frontal, mid-frontal, central and mid-central electrodes. The electrode sites for analysis were chosen based on scalp maps that showed intense activation in these regions. Similar methods for electrode grouping were used in previous ERP studies [57,58,59,60]. The frontal electrodes included F3, F5, F7, FC3, FC5, FT7 and the corresponding electrodes on the right hemisphere. The central electrodes included T7, TP7, C3, C5, CP3, CP5 and the corresponding electrodes on the right hemisphere. The midline frontal electrodes included F1, Fz, F2, FC1, FCz and FC2. The midline central electrodes included C1, Cz, C2, CP1, CPz and CP2. P300 analysis was conducted for central, mid-central, parietal and mid-parietal sites. The parietal electrodes included P3, P5, P7, PO3, PO5, PO7 and the corresponding right-hemisphere electrodes. The midline parietal electrodes included P1, Pz, P2 and POz. All the ERP measures from the 11 subjects were subjected to repeated-measures ANOVA with the ear and condition as independent variables.

## 3. Results

### 3.1. OAE Data

**Figure 1:**
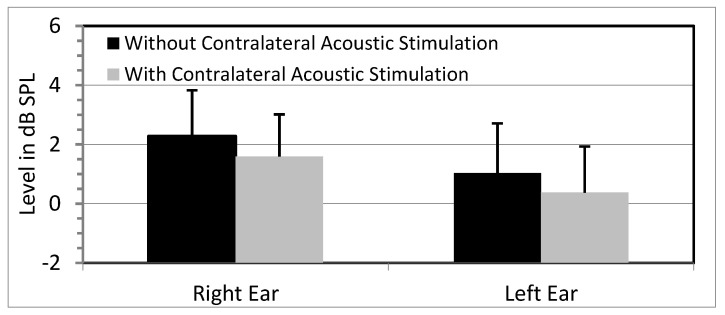
Mean overall amplitudes of click-evoked otoacoustic emissions (OAEs) for the two conditions: (1) without contralateral acoustic stimulation (CAS) and (2) with CAS in the two ears. Error bars indicate standard errors.

Click-evoked OAEs were present at acceptable SNRs in both ears for all the conditions, and the probe stability was always close to 100%. The average amplitudes of the click-evoked OAEs were compared for conditions with and without contralateral masking noise. The mean overall amplitudes for the right and left ears are shown in Figure 1. The amplitude data from the two ears were analyzed using repeated-measures ANOVA with the ear and condition as independent variables. There was a significant ear effect (F(1,10) = 6.68; *p* = 0.03, η_p_^2^ = 0.40). A main effect of condition was also seen (F(1,10) = 6.87; *p* = 0.03, η_p_^2^ = 0.41), with lower amplitudes in the presence of CAS. The interaction between the ear and noise was not significant (F(1,10) = 0.02; *p* = 0.91, η_p_^2^ = 0.002).

### 3.2. WRS Data

The word recognition scores for the NST calculated for the conditions with and without CAS are presented in Table 2. The main effects (ear: F(1,10) = 0.17; *p* = 0.67, *η_p_*^2^ = 0.02; condition: F(1,10) = 1.79; *p* = 0.21, *η_p_*^2^ = 0.15) and interaction (ear × interaction: F(1,10) = 0.25; *p* = 0.63, *η_p_*^2^ = 0.02) were non-significant.

### 3.3. MMN Data for Passive Listening Condition

**Figure 2:**
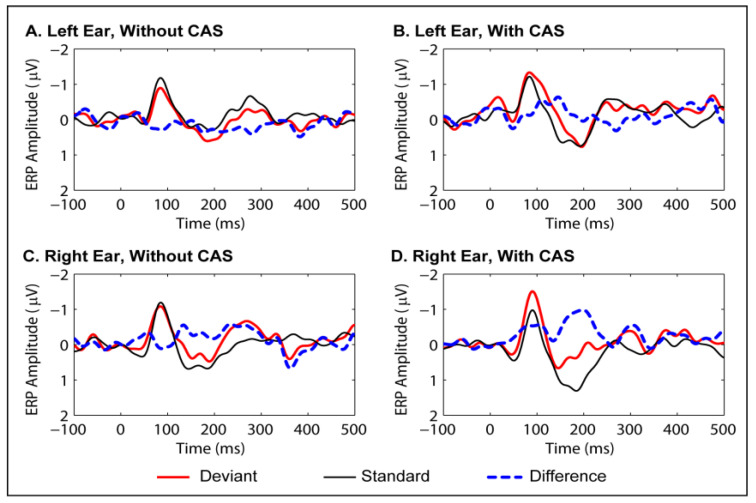
Grand average waveforms for event-related potential (ERP) responses to standard and deviant stimuli in the passive (MMN) condition, and the difference between the two (deviant–standard). Waveforms were averaged across frontal, mid-frontal, central and mid-central sites. Panel (**A**): left ear, no contralateral acoustic stimulation (CAS). Panel (**B**): left ear, with CAS. Panel (**C**): right ear, no CAS. Panel (**D**): right ear, with CAS.

Grand average waveforms of the MMNs obtained for the two conditions are presented in Figure 2. A repeated-measures ANOVA was performed after selecting electrodes from the frontal, mid-frontal, central and mid-central locations of the scalp, where the MMN was robust. The latencies and amplitudes were subjected to separate repeated-measures ANOVAs with condition and electrode location as factors. Descriptive statistics of the MMN are presented in Table 3, and the repeated-measures ANOVA results, in Table 4. A significant main effect of ear was seen for the MMN latencies, with peak latencies longer for the left ear than for the right ear. The other main effects and interactions were non-significant.

### 3.4. P300 Data for Active Listening Condition

**Figure 3:**
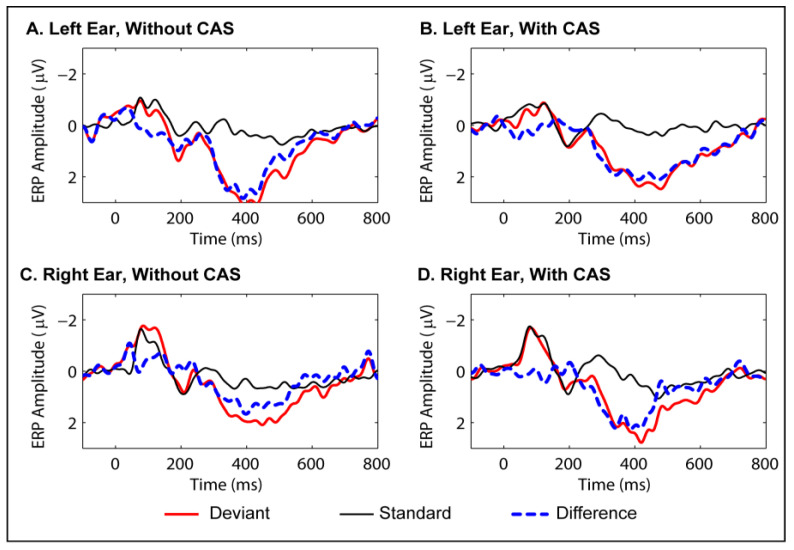
Grand average waveforms for ERP responses to standard and deviant stimuli in the active discrimination (P300) condition and the difference between the two (deviant–standard). Waveforms were averaged across central, mid-central, parietal and mid-parietal sites. Panel (**A**): left ear, no contralateral acoustic stimulation (CAS). Panel (**B**): left ear, with CAS. Panel (**C**): right ear, no CAS. Panel (**D**): right ear, with CAS.

Grand average waveforms of the P300s obtained for the two conditions are presented in Figure 3. Descriptive statistics are presented in Table 5. A repeated-measures ANOVA was performed after selecting the central, mid-central, parietal and mid-parietal scalp locations where the P300 was most prominent, and the results are shown in Table 6. Once again, the condition without contralateral noise was compared with the condition with contralateral noise. The Peak P300 latencies and peak amplitudes did not show significant differences between the two conditions.

The criterion and d’ were measured from the hits and false alarms obtained in the active discrimination (P300) task. The reaction times from the two conditions (with and without CAS) were also subjected to statistical analysis. Descriptive statistics for the d’, criterion and reaction times are shown in Table 7. None of the measures showed significant differences (Table 8).

## 4. Discussion

This exploratory study attempted to address an important gap in our understanding of the function of the auditory MOC efferent system in specific listening conditions by assessing how contralateral noise affected behavioral and physiological measures. The effects of efferent suppression on OAE amplitude and word recognition in noise were measured along with ERPs in passive and active tasks. For one physiological measure, namely the otoacoustic emissions elicited by clicks presented in quiet, contralateral noise decreased the level. For the other physiological and behavioral measures, stimuli were presented in ipsilateral noise with and without contralateral noise, and no statistically significant effect of contralateral noise was seen in any condition.

### 4.1. OAE Inhibition

In this study, click-evoked OAEs were analyzed in the 1000 Hz to 4000 Hz frequency range, where maximum suppression is seen in humans [61,62,63,64]. Animal studies also reveal that mid-to-high frequencies are strongly affected by the activation of the MOC system [11]. The overall amplitudes of the click-evoked OAEs were reduced in the presence of CAS, which is consistent with previous findings, exhibiting an inhibitory effect at the cochlear level [4,45]. The differences in OAE levels were seen as an ear effect, with the right ear showing higher levels compared with the left. The ear effect may be attributed to the asymmetry between the two ears [65], which could be related to handedness [66]. In our study, all the participants were right-handed.

### 4.2. Word Recognition Testing

In the present study, the nonsense-syllable recognition scores in noise from the nonsense syllable test were unchanged in the presence of CAS. This contrasts with previous findings obtained using similar methodology but with words that carried semantic content. Studies of speech perception have used monosyllables in noise in the past [23,24]. When nonsense syllables are used, listeners have to rely solely on acoustic features due to the lack of linguistic context. These results suggest that the antimasking effect alone is insufficient to lead to improvement in word recognition scores in adults.

In a 2012 study by de Boer et al. [22], phoneme discrimination was uncorrelated with OAE inhibition. Similarly, in another study [15], once subjects had been sufficiently trained in phoneme discrimination, the correlation between OAE inhibition and phoneme discrimination was lost, suggesting a stronger role for attention and/or other central mechanisms. The fact that several other studies have failed to show a relationship between word recognition scores and OAE inhibition may be attributed to differences in the methods used to test MOC effects on word recognition [27]. Modeling of efferent effects [67] shows that optimal speech perception is achieved when the amount of efferent activity is proportional to the level of noise, with the amount of unmasking dependent on both the signal level and noise level. It has also been shown that individuals with stronger MOC efferent responses are more responsive to changes in the SNR [68]. Therefore, it seems that a specific combination of signal level and noise level is required to achieve maximal improvement. Additionally, improvements may be age related [24], with young participants showing greater reliance on efferent function in noise. Those who have received a vestibular neurectomy show nearly normal performance, which points to the role of possible compensatory mechanisms in word recognition [26,66]. In summary, it is improbable that the MOC effect on the cochlea induced using CAS alone at a single SNR would appreciably affect speech intelligibility in ipsilateral noise at the group level.

### 4.3. ERPs

Although significant OAE inhibition was seen due to contralateral acoustic stimulation, the MMN (passive task) showed no statistically significant difference between the conditions with and without CAS. Obligatory changes that facilitate signal processing in noise can be measured in the cochlea and the cochlear nerve (in terms of changes to cochlear gain and the enhanced coding of signals in noise, respectively), but these may not be reflected at the cortical level in the MMN response. Alternatively, perhaps the changes are reflected at the cortical level but are sufficiently subtle that the MMN was not sensitive enough to capture them—at least when using these particular sound levels. Interestingly, an ear effect was noted for the MMN peak latency, with the latencies for the stimuli presented to the left ear longer than the latencies for the stimuli presented to the right ear. This latency difference suggests longer processing times for stimuli in the left ear. Ear differences have been reported in MMN amplitudes in response to monaural stimulation, especially in patients with various cortical lesions [69]. Interestingly, the left ear was also where the average d’ wsa highest in the P300 behavioral task.

The P300, which is reflective of conscious perception, was not shown by the ANOVA results to be significantly affected by the presence or absence of CAS. Behavioral performance was also essentially identical at the group level regardless of whether CAS was present. Once again, although a peripheral facilitatory mechanism is likely to be operating, its effects were not observed at the cortical level in the presence of attentional processing. As with MMN, the possibility remains that the P300 was simply not sensitive enough to capture the subtle changes transmitted from the cochlear mechanism. Interestingly, the ANOVA of the behavioral results showed a significant ear effect, with d’ being higher when stimuli were presented to the left ear rather than the right. It is possible that the participants were expending greater effort when stimuli were being presented to the left ear, with consequent gains in behavioral outcomes.

Evidence from others [22,29] points to a dynamic relationship between MOC function and central mechanisms during tasks in which individuals are attending to signals in noise. Behavioral output is modulated by interactions between the MOC system and central mechanisms that are attention- and experience-dependent. Indeed, long-term training was found to preserve performance in a signal-in-noise sound localization task in cats with MOC lesions. The authors attributed this to the development of alternate listening strategies, which were able to minimize the functional consequences of the auditory lesions [70]. Therefore, central mechanisms may compensate for significant changes in peripheral function in the processing of complex signals involving redundancy (e.g., speech) or during highly routine tasks (e.g., the psychophysical testing of intensity or frequency discrimination). Alternately, the unmasking provided by the MOC system may not be used at all [16,25]. It has been shown that the efferent system may have a greater role to play during auditory development in early childhood, when the central mechanisms for listening in noise are still immature [29]. The ERP data from the present adult study also support the hypothesis that passive and active central mechanisms may not reflect the facilitatory changes recorded at the periphery in laboratory conditions.

### 4.4. Methodological Challenges and Lessons

All the physiological measures for our study were obtained with stimuli ideal for eliciting a particular type of response while maintaining the general characteristics of the protocol. For example, transient tone bursts were embedded in white noise to elicit ERPs, and clicks were used to elicit OAEs to record OAE inhibition. Attentional focus was dependent on the task. In the OAE task, the participants were asked to be quiet and relax as fully as possible. In the passive MMN task, the participants were watching a movie; during the active P300 task, the participants’ focus was on the ear being tested. This variation in methodology was inevitable due to the range of physiological indices used. A frequency discrimination task was used to elicit ERPs, as it is an important skill for speech perception in noise [71]. Additionally, the frequency discrimination thresholds of the second formant frequency are reportedly affected by the lesioning of the MOC system in cats [65]. However, we need to acknowledge the possibility that the stimuli and presentation levels chosen, including for the contralateral noise, may be unnatural and work against the original hypotheses. ERP measurements require a considerable amount of the participant’s time, and we had four conditions even without multiple signal levels. Given the time constraint, we chose the most frequently reported SNR in the literature that showed enhancement with syllables or speech. For each condition in our study, only one level for the stimulus and the ipsilateral noise was chosen, which may not have been the optimal levels for demonstrating the associations between MOC effects and central auditory processing [72]. This is particularly important given the fact that the ipsilateral noise would also be evoking the MOC reflex in the presence of considerable within- and across-subject variabilities upon contralateral acoustic stimulation [73].

Another possible limitation is the methodology used to measure OAE inhibition [74]. Stimulus-frequency OAEs (SFOAEs) have been proposed to be superior to click-evoked OAEs in eliciting OAE efferent inhibition, given that the signal levels required to elicit click-evoked or tone-evoked OAEs may themselves cause MOC activation. SFOAEs may be elicited with continuous tones at lower stimulus intensities than click-evoked OAEs. However, commercial systems that measure SFOAEs are not currently available. The subclinical activation of the middle-ear muscle reflex could contaminate MOC reflex effects. We present the caveat that due to the signal levels used in this study, although unlikely, it is possible that the middle-ear reflex may have contaminated the results obtained from couple of participants with low middle-ear reflex activation thresholds [75,76].

As the measures of OAE, word recognition in noise, MMN and P300 were obtained in different test sessions with different sets of stimuli and listening conditions, involving considerable amounts of test time for each of our participants, it is methodologically challenging to test many different signal levels to find out what may be sensitive to the interactions between MOC activity and higher-level cortical processing when listening in noise. Given that the MOC efferent system works both ipsilaterally and contralaterally with much of the innervation being ipsilateral, we cannot rule out the possibility that the ipsilateral MOC could have been fully activated in our behavioral experiment, providing enhanced listening-in-noise recognition. Presumably, this should apply whether or not contralateral noise stimulation was applied. Likewise, if the ipsilateral MOC has achieved full activation in the EEG experiments for delivering the hypothesized anti-masking benefits, there is no reason to expect any additional benefit from adding contralateral noise. Therefore, our conditions may represent a degree of MOC activation (less activation with ipsilateral noise only vs. more activation with added contralateral noise stimulation) as opposed to conditions with vs. without efferent activation. In experiments with human subjects, it is methodologically challenging to implement protocols for introducing a condition with no MOC activation for comparison to verify the contributions of MOC to listening in noise [2].

Changes have been reported in ERPs in the presence of ipsilateral noise compared with a quiet condition [33,34,38,39,77,78,79,80]. Generally, amplitudes are reduced and latencies are prolonged for the MMN and P300 when stimuli are presented in ipsilateral noise, which may be activating the efferent system. As we did not include a quiet condition for comparison, we were unable to capture this effect.

Future studies may need to consider simultaneous experimental protocols that can measure attentional modulation effects in the periphery as well as at the cortical level in the same test session [81]. Nevertheless, the exploratory results reported here could provide a cautionary note to avoid simplistic expectations or interpretation with regard to cortical and medial efferent systems in auditory perception [25]. For instance, one should not erroneously assume that due to the MOC “antimasking” effect, presenting contralateral noise at a single predetermined “optimal” SNR would automatically lead to enhanced ipsilateral speech-in-noise performance. The relationship of MOC efferent activity with higher-level auditory and speech processing is highly dependent on the task characteristics, including the SNRs [72].

### 4.5. Clinical Relevance and Future Directions

In many clinical populations exhibiting difficulties with speech perception in noise (for example, individuals with learning disabilities and children with auditory processing disorders), inadequate functioning of the MOC system has been documented [65,82,83]. Based on the results of this study, it can be hypothesized that inefficient MOC function is just one piece of the puzzle in these individuals who have not been able to develop alternative listening strategies, as normal individuals do, to compensate for deficiencies in MOC function.

Interestingly, increased MOC activity has been documented in musicians [84]. Greater OAE inhibition has been found in musicians compared with age- and gender-matched normal subjects. Evidence suggests that musicians have superior behavioral speech-in-noise processing skills and brainstem coding [85,86]. An interesting hypothesis is that the enhanced auditory experience in musicians leads to a cumulative strengthening of these mechanisms.

Future studies should be directed toward understanding the role of the MOC system and its interactions with attention, learning and speech perception. Furthermore, information is required about its role during the developmental period [29] and in clinical populations [87,88]. This will inform the optimal procedures to use for the testing and rehabilitation of those who exhibit speech-in-noise difficulties.

## 5. Conclusions

In summary, this auditory perception study implemented different behavioral and neurophysiological protocols that involved varying degrees of MOC activation. OAE inhibition was seen in the presence of contralateral acoustic stimulation. The recognition of nonsense syllables did not appear to change with the degree of MOC stimulation. Similarly, cortical ERPs as assessed by the MMN and P300 responses in passive and active listening conditions did not reflect the facilitatory effects seen in the contralateral inhibition of OAE at the cochlear level. Our findings are consistent with the view that individuals do not necessarily make use of the available MOC induced unmasking mechanisms for higher-level auditory processing and speech perception in noise, which are highly subject to influence from age-dependent attentional, cognitive and experiential factors [25]. The dissociation patterns demonstrate the limitations of the materials and methods implemented in the present study, which underlines the need for further studies to address the complexity and challenge with suitable protocols to reveal the possible associations and interactions between cortical and MOC efferent mechanisms in auditory perception.

## Figures and Tables

**Table 1:** Average audiometric thresholds (in dB Hearing Level (HL)) and standard deviations (within parentheses) at different octave frequencies in the two ears for participants in the study.

Ear	250 Hz	500 Hz	1000 Hz	2000 Hz	4000 Hz	8000 Hz
**Right**	5 (4.9)	4.2 (3.9)	2.3 (4.7)	2.3 (7.5)	3.8 (7.2)	2.6 (7.8)
**Left**	5 (8.3)	4.6 (5.7)	3.5 (4.3)	2.3 (7.9)	1.9 (6.1)	2.3 (8.5)

**Table 2:** Descriptive statistics of word recognition scores without and with contralateral acoustic stimulation (CAS) in the two ears. SD = Standard Deviation.

Ear	Condition	Word Recognition Score in Percentage (SD)
Right	Without CAS	78.3 (3.6)
	With CAS	76.8 (3.7)
Left	Without CAS	77.4 (5.2)
	With CAS	77 (3.3)

**Table 3:** Descriptive statistics of peak amplitudes and peak latencies of mismatch negativity (MMN) in the two conditions in the two ears. Please see text for sites grouped for analysis. CAS = Contralateral Acoustic Stimulation, µV = micro Volts, ms = milliseconds, SD = Standard Deviation.

Ear	Condition	MMN	
		Amplitude (µV) (SD)	Latency (ms) (SD)
Right	Without CAS	−1.22 (0.47)	227.3 (23.5)
	With CAS	−1.15 (0.34)	219.65 (18.63)
Left	Without CAS	−1.21 (0.33)	247.72 (20.93)
	With CAS	−1.1 (0.52)	228.95 (18.84)

**Table 4:** Repeated-measures ANOVA results for MMN amplitude and latency. (* stands for *p* < 0.05).

Main Effects and Interactions	MMN Peak Amplitude	MMN Peak Latency
Ear	*F*(1,10) = 0.145 *p* = 0.71 η_p_^2^ = 0.014	*F*(1,10) = 6.71 *p* = 0.03 * η_p_^2^ = 0.40
Condition	*F*(1,10) = 1.25 *p* = 0.29 η_p_^2^ = 0.11	*F*(1,10) = 2.11 *p* = 0.17 η_p_^2^ = 0.175
Ear × Condition	*F*(1,10) = 0.058 *p* = 0.81 η_p_^2^ = 0.006	*F*(1,10) = 1.4 *p* = 0.26 η_p_^2^ = 0.263

**Table 5:** Descriptive statistics of peak amplitudes and peak latencies of MMN and P300 in the two conditions in the two ears. Please see text for sites grouped for analysis. CAS = Contralateral Acoustic Stimulation, µV = micro Volts, ms = milliseconds, SD = Standard Deviation.

Ear	Condition	MMN		P300	
		Amplitude (µV) (SD)	Latency (ms) (SD)	Amplitude (µV) (SD)	Latency (ms) (SD)
Right	Without CAS	−1.22 (0.47)	227.3 (23.5)	2.09 (0.99)	420.5 (54.97)
	With CAS	−1.15 (0.34)	219.65 (18.63)	1.8 (0.95)	417.03 (64.5)
Left	Without CAS	−1.21 (0.33)	247.72 (20.93)	1.99 (0.98)	409.52 (49.83)
	With CAS	−1.1 (0.52)	228.95 (18.84)	1.97 (0.98)	444.12 (50.45)

**Table 6:** Results of repeated-measures ANOVA for P300 peak amplitude and peak latency.

Main Effects and Interactions	P300 Peak Amplitude	P300 Peak Latency
Ear	*F*(1,10) = 0.022 *p* = 0.88 η_p_^2^ = 0.002	*F*(1,10) = 0.72 *p* = 0.416 η_p_^2^ = 0.067
Condition	*F*(1,10) = 1.20 *p* = 0.298 η_p_^2^ = 0.107	*F*(1,10) = 0.85 *p* = 0.378 η_p_^2^ = 0.078
Ear × Condition	*F*(1,10) = 0.533 *p* = 0.482 η_p_^2^ = 0.051	*F*(1,10) = 1.65 *p* = 0.228 η_p_^2^ = 0.142

**Table 7:** Descriptive statistics of d’ (sensitivity), criterion and reaction times obtained in the active-discrimination condition (P300 task) without and with contralateral acoustic stimulation (CAS) in the two ears. SD = Standard Deviation.

Ear	Condition	d’ (SD)	Criterion (SD)	Reaction Times (SD)
Right	Without CAS	2.07 (1.12)	−0.69 (0.38)	556.01 (115.66)
	With CAS	2.09 (1.18)	−0.76 (0.34)	567.29 (134.59)
Left	Without CAS	2.59 (1.34)	−0.66 (0.5)	543.18 (121.04)
	With CAS	2.64 (1.42)	−0.67 (0.43)	551.19 (110.43)

**Table 8:** Results of repeated-measures ANOVA for d’ (sensitivity), criterion and reaction times obtained in the active listening condition (P300 task). (* stands for *p* < 0.05).

Main Effects and Interactions	d’	Criterion	Reaction Time
Ear	*F*(1,10) = 7.133 *p* = 0.02 * η_p_^2^ = 0.41	*F*(1,10) = 0.702 *p* = 0.42 η_p_^2^ = 0.06	*F*(1,10) = 1.24 *p* = 0.292 η_p_^2^ = 0.11
Condition	*F*(1,10) = 0.62 *p* = 0.80 η_p_^2^ = 0.006	*F*(1,10) = 1.81 *p* = 0.208 η_p_^2^ = 0.153	*F*(1,10) = 0.420 *p* = 0.53 η_p_^2^ = 0.04
Ear × Condition	*F*(1,10) = 0.121 *p* = 0.735 η_p_^2^ = 0.012	*F*(1,10) = 0.708 *p* = 0.420 η_p_^2^ = 0.066	*F*(1,10) = 0.015 *p* = 0.91 η_p_^2^ = 0.001

## Abbreviations

The following abbreviations are used in this manuscript:

ANOVA	Analysis of variance
BBN	Broadband noise
CAS	Contralateral acoustic stimulation
CID	Central Institute for the Deaf
ER	Etymotic research
ERP	Event-related potentials
MOC	Medial olivocochlear
MMN	Mismatch negativity
NST	Nonsense Syllable Test
P300	Positive peak at 300 ms
OHC	Outer hair cell
OAE	Otoacoustic emission
SL	Sensation level
SPL	Sound pressure level
SNR	Signal-to-noise ratio
SRT	Speech reception threshold
TEOAE	Transient-evoked otoacoustic emissions
WRS	Word recognition score
